# Synchrotron CT dosimetry for wiggler operation at reduced magnetic field and spatial modulation with bow tie filters

**DOI:** 10.1107/S1600577524008531

**Published:** 2024-10-22

**Authors:** Stewart Midgley, Nanette Schleich, Andrew Stevenson, Alex Merchant

**Affiliations:** a Medical Physics and Radiation Engineering Canberra Hospital Yamba Drive Garran ACT2605 Australia; b Department of Radiation Therapy, University of Otago, Wellington, New Zealand; c ANSTO/Australian Synchrotron, 800 Blackburn Road, Clayton, VIC3168, Australia; d CSIRO Manufacturing, Private Bag 10, Clayton South, VIC3169, Australia; e Barwon Health, PO Box 281, Geelong, VIC3220, Australia; Tohoku University, Japan

**Keywords:** Australian Synchrotron Imaging and Medical Beamline, wiggler magnetic field strength, third harmonic, bow tie filter, CT dosimetry

## Abstract

The Australian Synchrotron Imaging and Medical Beamline (IMBL) uses a superconducting multipole wiggler (SCMPW) source, dual crystal Laue monochromator and 135 m propagation distance to enable imaging and computed tomography (CT) studies of large samples with mono-energetic radiation. This study aimed to quantify two methods for CT dose reduction: wiggler source operation at reduced magnetic field strength, and beam modulation with spatial filters placed upstream from the sample. Transmission measurements with copper were used to indirectly quantify the influence of third harmonic radiation. Operation at lower wiggler magnetic field strength reduces dose rates by an order of magnitude, and suppresses the influence of harmonic radiation, which is of significance near 30 keV. Beam shaping filters modulate the incident beam profile for near constant transmitted signal, and offer protection to radio-sensitive surface organs: the eye lens, thyroid and female breast. Their effect is to reduce the peripheral dose and the dose to the scanned volume by about 10% for biological samples of 35–50 mm diameter and by 20–30% for samples of up to 160 mm diameter. CT dosimetry results are presented as in-air measurements that are specific to the IMBL, and as ratios to in-air measurements that may be applied to other beamlines. As CT dose calculators for small animals are yet to be developed, results presented here and in a previous study may be used to estimate absorbed dose to organs near the surface and the isocentre.

## Abstract 2

Operation at lower wiggler magnetic field strength reduces dose rates by an order of magnitude, and suppresses the influence of harmonic radiation, of significance near 30 keV. Beam shaping filters modulate the incident beam for near constant transmitted signal and offer protection by reducing the average dose to the scanned volume at 30–100 keV by about 10% for biological samples of 35–50 mm in diameter and by 20–30% for samples of up to 160 mm in diameter.

## 1. Introduction

The Australian Synchrotron Imaging and Medical Beamline (IMBL) uses a superconducting multipole wiggler (SCMPW) to enable high dose rate radiobiology studies (hutches 1B and 2B), imaging and computed tomography (CT) with large samples (hutch 3B). The beam size is approximately 10 mm × 2 mm at hutch 1B (22 m from the source), 100 mm × 3 mm at hutch 2B (35 m from the source) and over 500 mm × 30 mm at 3B (135 m from the source). As synchrotron CT studies can expose the sample and image receptor to significant amounts of radiation, we report dosimetry measurements and investigate strategies for radiation dose reduction. A dual crystal Laue monochromator (DCLM) delivers near mono-energetic radiation and reduces the dose rate in air by two or more orders of magnitude. CT instrumentation uses a sample rotation stage and stationary image receptor achieving spatial resolution in the range 10–100 µm. At the time of these experimental measurements (2019), IMBL CT studies focused more on *ex vivo* specimens and non-biological samples, with scan durations of tens to hundreds of seconds delivering dose in air at the sample stage of the order of Grays or more.

The DCLM (see Fig. 10 of Stevenson *et al.*, 2017 ▸) is fabricated in silicon with a diamond face-centred cubic Bravais lattice, with crystallographic selection rules allowing reflections for Miller indices (*h*, *k*, *l*) all odd, or all even with *h* + *k* + *l* = 4*n*. The Bragg equation passes the fundamental reflection Si 111, and the third order (Si 333 reflection) is the first significant harmonic as the Si 222 reflection is ‘forbidden’. The presence of harmonics can lead to beam hardening artefacts whereby the lower energy fundamental is preferentially removed, and the beam becomes more penetrating. This is manifested in the CT reconstruction as systematic errors with reduced attenuation coefficients at the centre of a homogeneous object (cupping artefact), and dark streak artefacts in directions where attenuation is strong (*e.g.* for high atomic number ‘foreign bodies’). Since diffraction peak intensity is proportional to wavelength, the dominant contribution is from the third harmonic whose influence increases for lower fundamental energies. Of interest is the lowest fundamental beam energy that can be considered to be mono-energetic. The absence of significant systemic errors due to beam hardening enables quantitative imaging techniques that exploit the compositional dependence of the photoelectric effect such as *K*-edge subtraction (Zhu *et al.*, 2014 ▸) and dual energy X-ray analysis (Midgley, 2013 ▸; Midgley & Schleich, 2015 ▸).

Medical imaging employs spatial modulation of the incident beam to compensate for thickness variations across the width of the sample (Bushberg *et al.*, 2012 ▸). Without modulation, the dynamic range for the image receptor and display device must span near 100% for the incident beam to about 0.5%, and surface organs receive significantly larger absorbed dose than those at depth. For over 100 years radiography has employed bolus materials (attached to the patient) or beam shaping (*e.g.* with triangular wedge filter) to compensate for thickness variations and deliver similar transmitted signal at the edge and centre of the sample. Synchrotron CT studies with bolus material attached to long and narrow fossil samples homogenizes the beam hardening artefacts to deliver improved reconstructed image quality (Sanchez *et al.*, 2013 ▸). In the 1970s, medical CT minimized the influence of beam hardening by placing a wedge on either side of the patient. The CT beam is also filtered to remove photons with energies below 30 keV. The EMI CT1010 scanner (New *et al.*, 1974 ▸) was the first commercial head CT system acquiring parallel beam scan data by translating the source and detector array across the sample, then repeating the process at different angles. The head was placed against a membrane inside a circular ‘water-box’ acting as the thickness compensating filter. Later CT generations with divergent fan beam geometry place a small ‘bow-tie’ filter close to the X-ray source. Such filters are small in size, fabricated from metals (Hseih, 2003 ▸; Mahesh, 2009 ▸; Kalender, 2011 ▸; Yang *et al.*, 2019 ▸) or use a liquid filled chamber (Liu *et al.*, 2014 ▸). Beam shaping filters deliver similarly transmitted spectra across the field of view with the same non-linear relationship between measured ray-sum (negative logarithm for the ratio of incident to transmitted signal) and sample thickness. This is characterized and corrected using a polynomial to transform measured to ‘ideal’ ray-sums that are proportional to thickness (Brooks & Di Chiro, 1976 ▸). A narrower range of transmitted signal allows better use of the available image receptor output dynamic range, whereby digitization rounding errors are reduced, leading to improved image quality. In addition, the absorbed dose near the surface is reduced to protect radio-sensitive organs (ICRP, 2007 ▸) such as the eye lens, thyroid and female breast.

The aims of this study were to quantify two strategies for reducing the radiation dose to the sample during synchrotron CT. We investigated the dose rate in air and beam quality for wiggler operation at ‘non-standard’ magnetic field strengths of 1.4–3.0 T. We evaluated the dose reduction afforded by spatial modulation with beam shaping filters suitable for near parallel beam synchrotron CT at the IMBL. Measurements considered phantoms representing a mouse, and small animal to human head sized subjects, complementing the previous report (Midgley *et al.*, 2019 ▸) without spatial modulation.

## 2. Materials and methods

### 2.1. IMBL source control and beam energy selection

Wiggler radiation is characterized by the critical energy representing the second quartile for the spectral distribution, proportional to magnetic field strength *B*, and radiated power proportional to *B*^2^ (Attwood, 2000 ▸). The lowest available mono-energetic photon energy is governed by the attenuation of beamline components as summarized in Table 1 ▸ for the IMBL (Stevenson *et al.*, 2017 ▸). The wiggler emits a substantial amount of power as non-ionizing radiation, absorbed by the *in vacuo* graphene and graphite filter materials. The minimum filter set F1 exposes the beamline components to thermal radiation emitted from these absorbers, blocked by the addition of aluminium (F2) and copper (F3). This study focuses on filter set F2.

**Figure 1:**
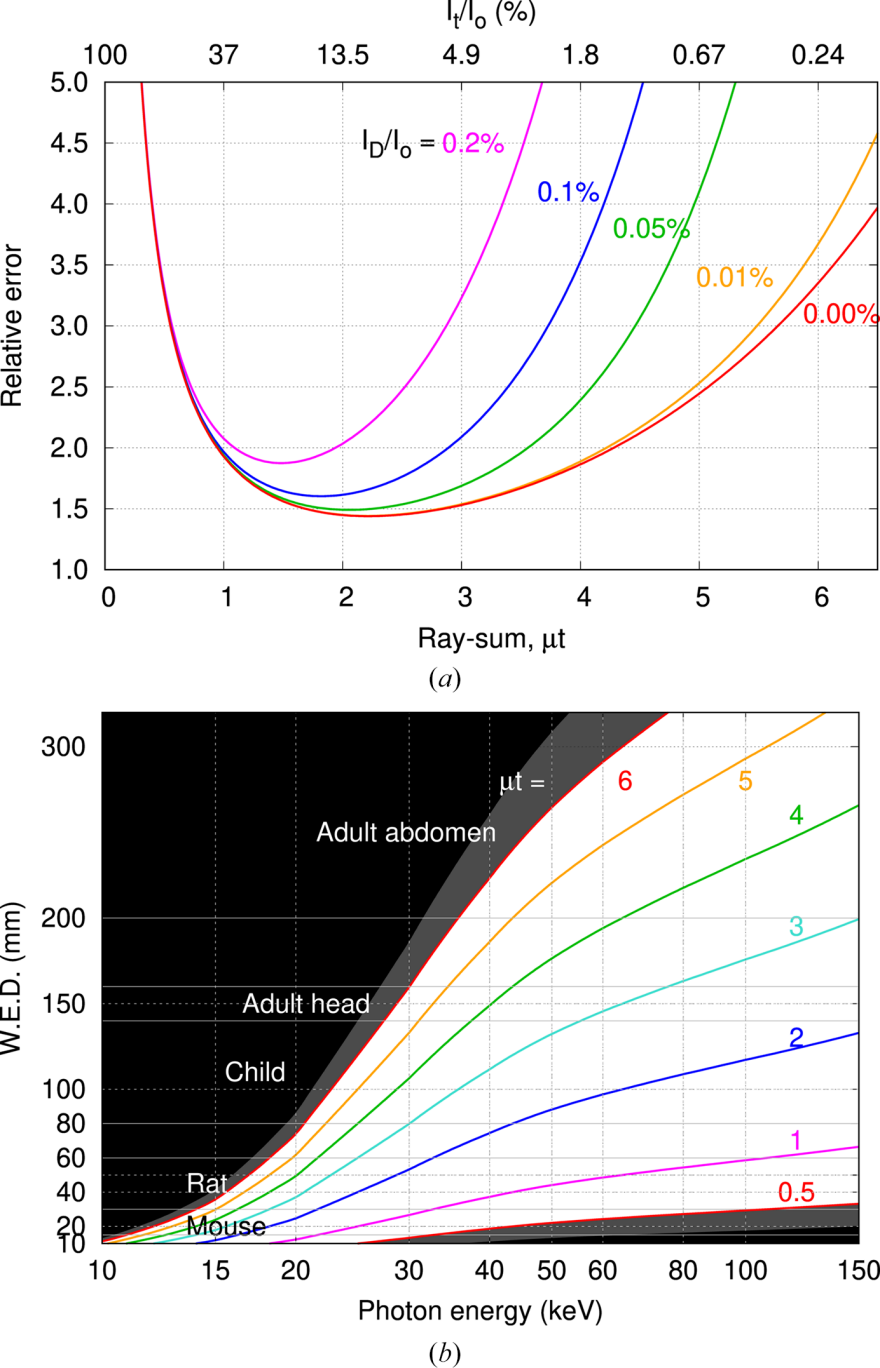
Propagation of error analysis for CT presented as (*a*) ray-sum uncertainty relative to that for the incident beam as a function of sample thickness, and the dark signal. Results are presented in (*b*) as the water equivalent diameter (WED) as a function of beam energy, also showing typical thickness ranges for CT studies.

Propagation of error analysis for transmission measurements (Rose & Shapiro, 1948 ▸) shows that noise in the CT reconstruction is amplified for very thin and very thick samples with a broad minimum for ray-sums 0.5 ≤ μ*t* ≤ 5.0 (Nördfors, 1960 ▸), where μ is the linear attenuation coefficient and *t* is thickness (also derived in the Appendix of Midgley, 2011 ▸). The influence of the dark signal *I*_D_, measured in the absence of radiation, is to reduce the upper ray-sum limit as illustrated in Fig. 1 ▸(*a*). The *y*-axis is the ratio of relative errors for ray-sums to those of the incident beam. Being greater than unity, this represents noise amplification for the ray-sum relative to that of the incident beam. For sample thickness μ*t* = 0.5–5.0, amplification is 1.5 to 3.0, and outside this range amplification becomes large. These results are presented in Fig. 1 ▸(*b*) for water filled samples as a surrogate for soft tissue. For example, a mouse with water equivalent diameter (WED) of 30 mm can be scanned at 15–100 keV, whilst larger samples require higher energies.

The standard magnetic field strengths for the IMBL SCMPW are 1.4, 2, 3 and 4 T. Changing from one field to another needs to be conducted by following a hysteresis curve from 0.94 to 1.4 to 2 to 3 to 4 to 4.2 T and then back to 0.94 T. Cycling around the full loop takes approximately an hour under normal operating conditions. Therefore, changing from 1.4 to 2 T, for example, takes only a few minutes, whereas changing from 2 to 1.4 T takes almost an hour. Changing wiggler field needs to be at a rate commensurate with limiting disturbance to the standard storage-ring operation (and thereby the other operating beamlines) but does sometimes result in dropping out of top-up mode, particularly for larger field changes. In the following, attention is restricted to the rocking curve peak representing the maximum available flux for the IMBL source and monochromator.

### 2.2. Quantification of harmonic radiation

The contribution from harmonic radiation (Ren *et al.*, 1999 ▸) was estimated indirectly from transmission measurements that were conducted at 25, 30 and 60 keV for wiggler field strengths of 1.4, 2.0 and 3.0 T. The IMBL Ruby camera (Hall *et al.*, 2013 ▸), with 43 µm-thick Gd_2_O_2_S:Tb screen, measured transmission through sheets of 100 mm × 100 mm electrolytic grade copper with thicknesses of 0.10–0.25 mm. At each beam energy, the copper sheets were arranged into eight steps spanning 0.5 ≤ μ*t* ≤ 4.0. Data collection acquired images with the beam shutter closed (dark field) and in the absence of the sample with the shutter open (flat field), repeated before and after recording transmission through copper. The average intensities of raw *I*_R_, dark *I*_D_ and flat *I*_F_ images were used to calculate the ray-sum, 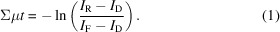
The dark signal pixel value was close to 100. Frame integration times were selected to keep average flat image pixel values within 50000–60000 without saturating the 16 bit camera, requiring 450, 40 and 80 ms at 25, 30 and 60 keV, respectively. Region of interest analysis used the software *ImageJ* (Schneider *et al.*, 2012 ▸). Measured ray-sums as a function of thickness were fitted to a spectral model with two components as described in the previous report, with third harmonic relative contribution of *f*/(1 + *f*).

### 2.3. CT dosimetry

The dose profile for CT studies is largest near the surface and decreases with depth. CT dose measurement indices were measured by integrating the radiation dose delivered during a CT scan with a dosimeter placed in air and at the centre and periphery of cylindrical phantoms (IMPACT, 1998 ▸, 2011 ▸; Bushberg *et al.*, 2012 ▸) fabricated from poly methyl methacrylate (PMMA). These are known as CT dose indices (CTDIs), and the in-air measurement represents the source brightness at the scanner’s rotation axis or isocentre. The volumetric CT dose index, CTDIvol, represents the absorbed dose to a soft tissue phantom averaged over the area of an axial slice, evaluated via 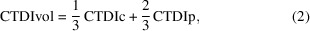
where CTDIc and CTDIp are the central and peripheral values, respectively. We used a PTW UNIDOS-E electrometer in conjunction with a PTW CTDI probe (model 30009), which is an air-filled pencil ionization chamber of 10 mm diameter by 100 mm active length with graphite walls and central electrode. The probe is calibrated for tungsten anode white radiation with beam qualities (IEC, 2005 ▸) of RQA8 to RQA10 at 100–150 kVp with half value layers (HVL) of 0.53–0.99 cm aluminium, which corresponds to mono-energetic beam energies of 50–80 keV. The calibration certificate (dated ten months before use) provides detector calibration factor *N*_*K*_ = 9.995 × 10^7^ Gy cm C^−1^ with probe correction factors of 0.99–1.02 and uncertainty ±3%. The electrometer correction factor is unity with ±0.5% uncertainty.

**Figure 2:**
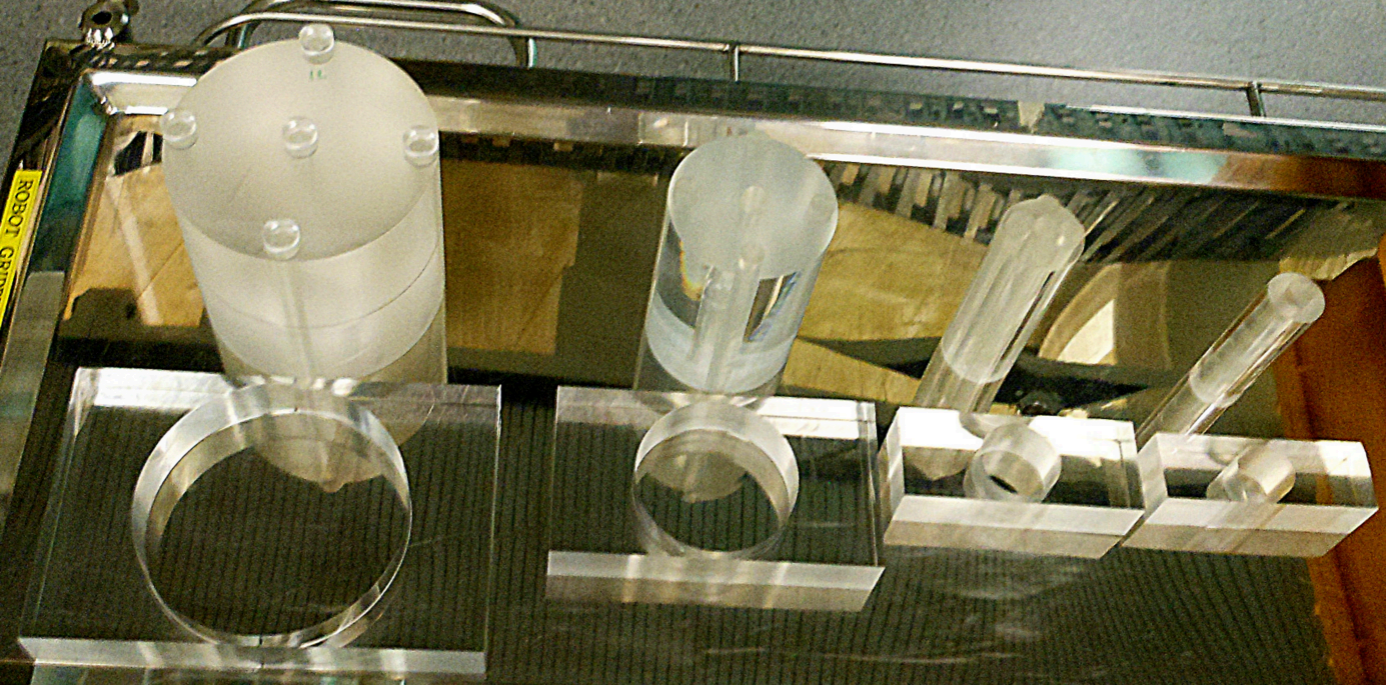
Custom built phantoms for measuring CT dose indices and their ‘bow tie’ filters, with dimensions (from left to right) of width × depth of 260 mm × 170 mm (filter void diameter *D* = 160 mm), 200 mm × 110 mm (*D* = 100 mm), 150 mm × 60 mm (*D* = 50 mm) and 135 mm × 45 mm (*D* = 35 mm).

The beam was collimated to 160 mm by 10 mm (width by height) for CTDI measurements in air, and 200 mm by 10 mm for measurements with the phantoms. CTDI was measured in air for three wiggler field strengths, 1.4, 2.0 and 3.0 T, and with the probe placed in different phantoms for 3.0 T only, recording 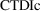 at the centre and 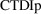 at the periphery. Measurements integrated over the duration of a 10 s CT scan involving 360° rotation and were repeated for beam energies of 25–100 keV. Head and body phantoms are commercially available for medical CT whilst the smaller items were designed and custom built for this study. The phantoms illustrated in Fig. 2 ▸ comprise 14 cm-long cylinders of PMMA with diameters of 35 mm (mouse body), 50 mm (rat body), 100 mm (child head) and 160 mm (child body or adult head). A 320 mm diameter body phantom was not used as this proved to be too heavy (14.5 kg) for the rotation stage. The mouse phantom has a single 12 mm-diameter hole at the centre. The larger phantoms have five holes of the same size, one at the centre and four at equal intervals around the periphery with their centres 10 mm below the cylinder surface. The holes are filled with PMMA rods, with one hole occupied by the CTDI probe for each measurement.

### 2.4. Beam shaping filters for synchrotron CT

Synchrotron CT at the IMBL involves a wide near-parallel beam of 10–30 mm height, so suitable ‘bow tie’ filters must have a width that is wider than the sample. Thus, for this study the beam shaping filters were designed as air-filled circular holes in a homogeneous slab of PMMA with diameters not smaller than the sample. Four items were constructed from 40 mm-thick rectangular blocks, with dimensions shown in Fig. 2 ▸, where the shortest path length is through the centre with remaining wall thickness of 5 mm at the front and rear each, resulting in a total of 10 mm of PMMA. Care was taken to polish any surfaces exposed to the beam to remove scratches that could otherwise be emphasized during propagation based phase contrast enhanced CT studies. The filters were placed 1 m upstream from the rotation stage and the image receptor was used to align the centres of the filter and the phantom with the vertical CT rotation axis. The CTDI measurements in air were conducted without the beam shaping filters.

## 3. Results

**Figure 3:**
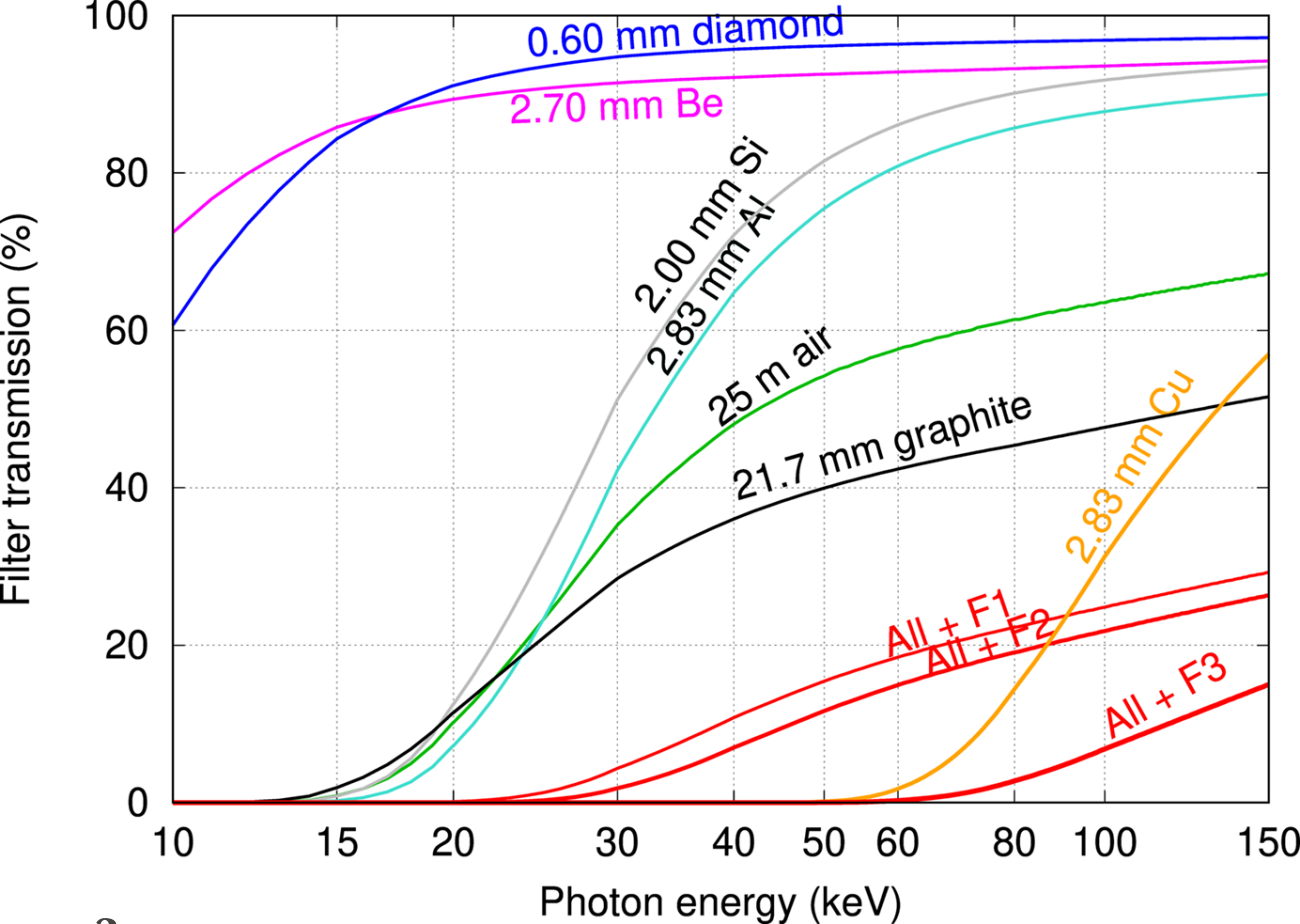
Transmission factors for beam windows, filters, optical components and room air evaluated using the NIST tabulation (Hubbell & Seltzer, 1995 ▸).

Attenuation for all beamline components (Table 1 ▸) is illustrated in Fig. 3 ▸ by means of energy dependent transmission factors. Dosimetry measurements used filter set F2, which prevents excessive thermal radiation reaching the beamline components, and in conjunction with the monochromator removes photon energies below 20 keV.

**Figure 4:**
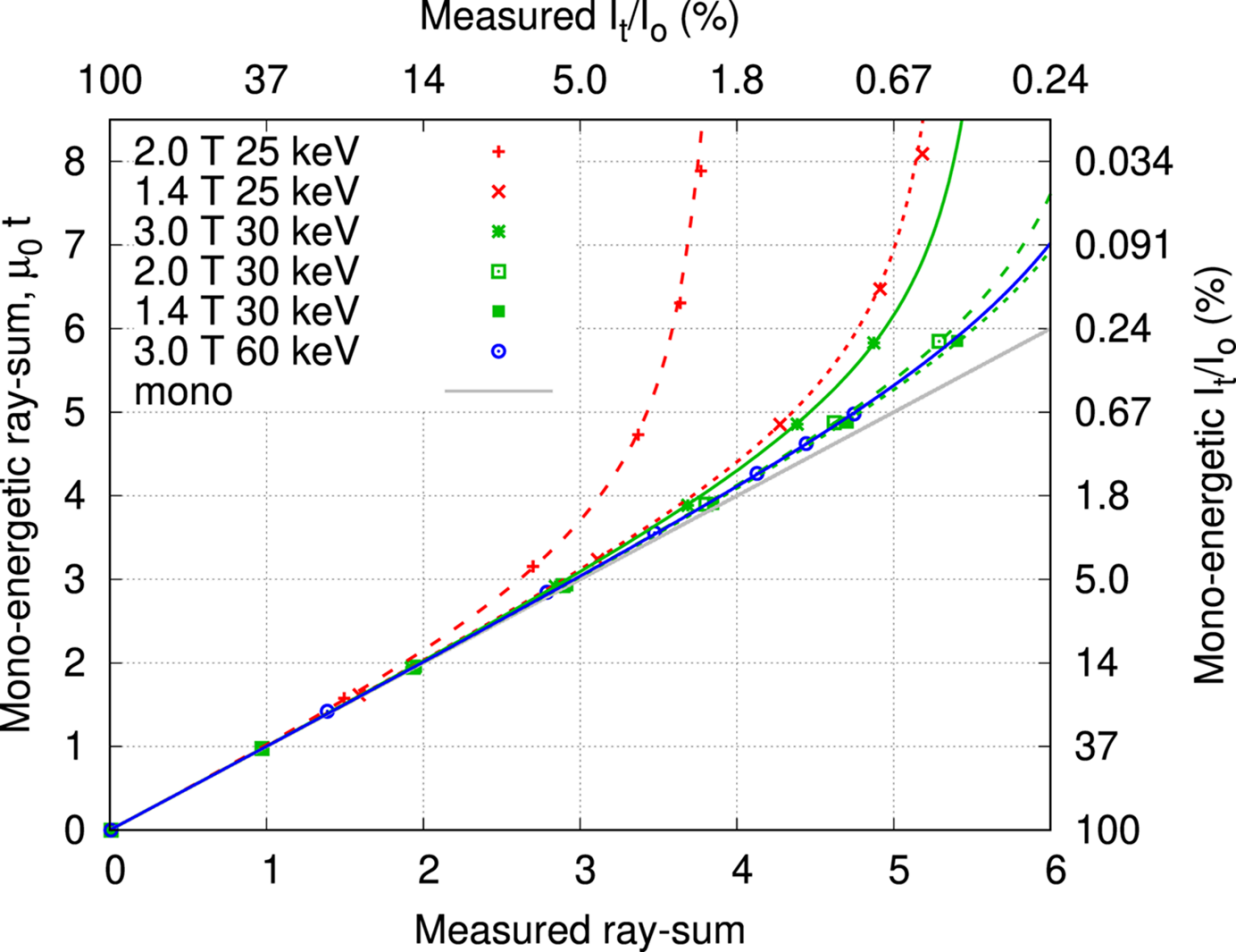
Transmission measurements for copper at 25–30 and 60 keV with wiggler field 1.4–3.0 T, also showing the ideal relationship for mono-energetic radiation.

**Figure 5:**
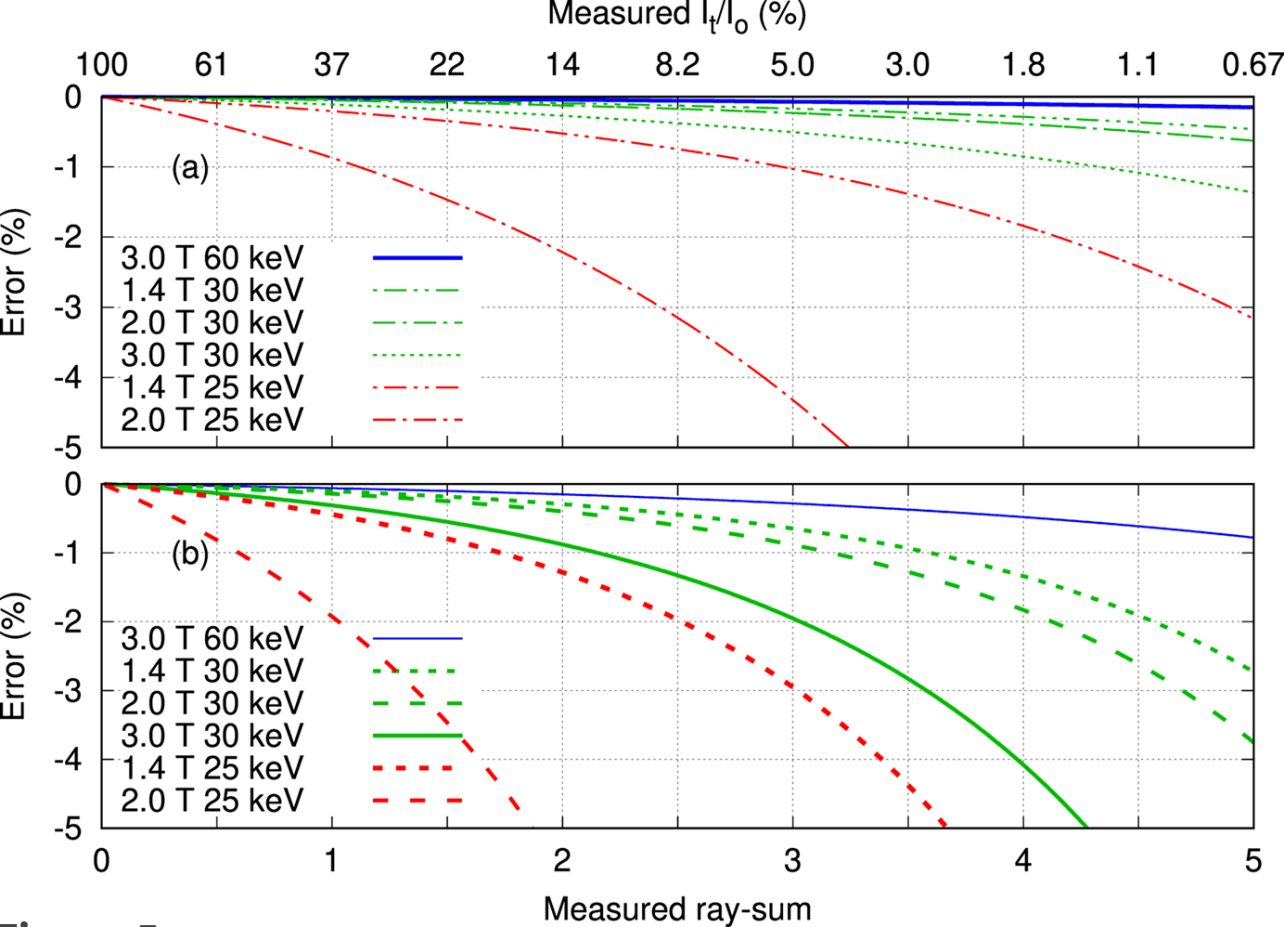
Predicted beam hardening errors for (*a*) water and (*b*) aluminium as a function of measured ray-sums, representing soft tissue and cortical bone.

Transmission measurement results for copper are presented in Fig. 4 ▸ as a function of beam energy and wiggler field strength, with fitted results for the third harmonic contribution *f* listed in Table 2 ▸. The difference between measurements and fitted model with fundamental and third harmonic offered no evidence of contribution from higher orders. The fitting programme reported uncertainties for fitted *f* (not presented) corresponding to 0.5–5% standard error of the mean (ratio of standard deviation to mean value). The two-component spectral model can be used to predict systematic errors for ray-sum measurements with other materials. Fig. 5 ▸ shows predictions for water and aluminium as surrogates for soft tissue and cortical bone. The *x*-axis is the measured poly-energetic ray-sum evaluated as −ln(*I*_t_/*I*_0_) and the *y*-axis is the relative difference against mono-energetic values, evaluated as the product of attenuation coefficient and thickness. Beam hardening leads to an underestimate with negative errors.

**Figure 6:**
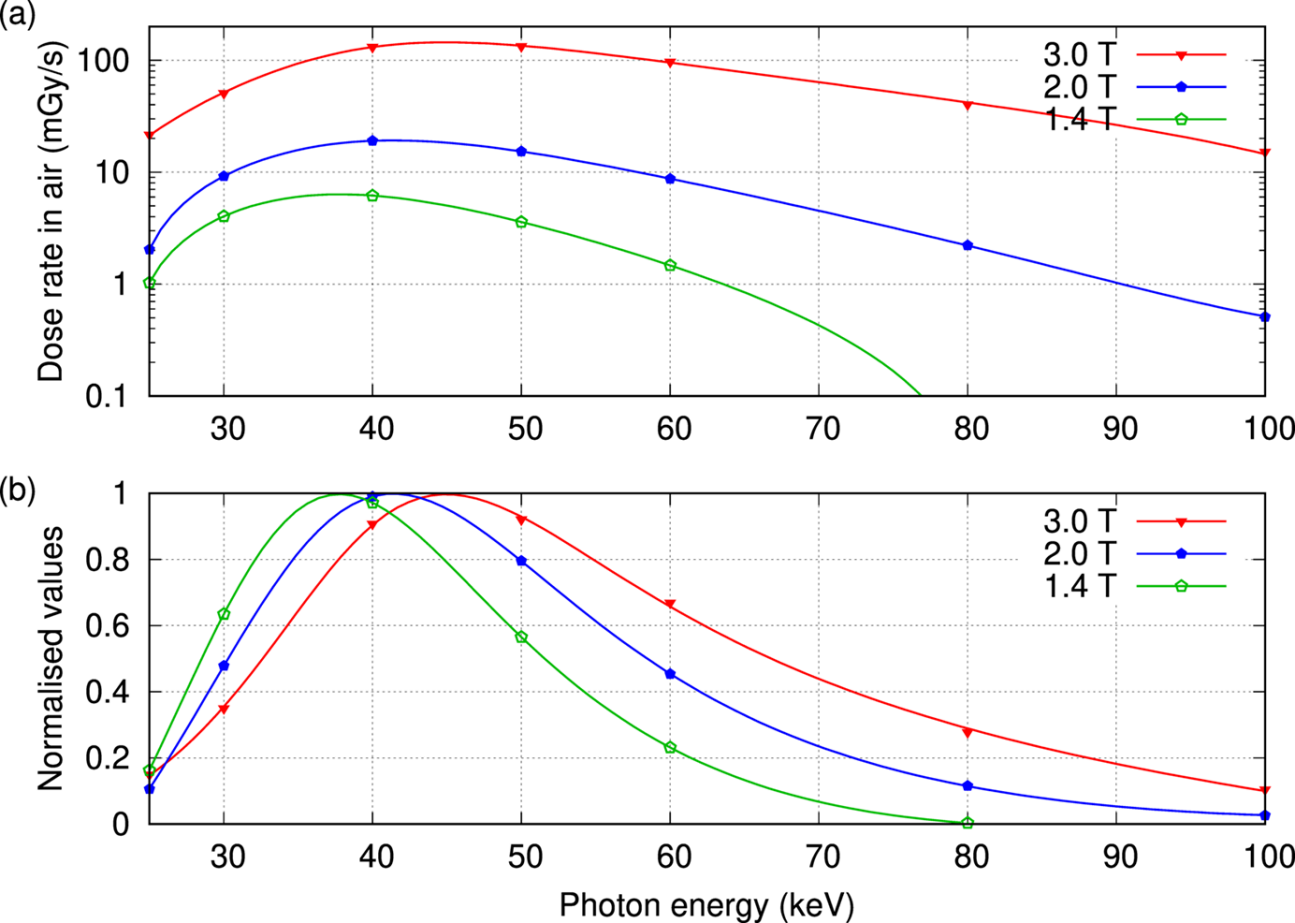
Dose rate in air for DCLM selected fundamental beam energies, (*a*) measured at the rocking curve peak, and (*b*) relative to the maximum for each wiggler field.

Fig. 6 ▸ shows the energy dependence for the mean dose rate in air averaged over the central region of the beam, which is defined by 10 mm horizontal active diameter for the CTDI probe by 10 mm vertical collimation. Curves were fitted using *gnuplot* (Williams & Kelley, 2011 ▸) to ratios of third to second degree polynomials; the spectral shape was emphasized by normalization to the same peak value in Fig. 6 ▸(*b*).

**Figure 7:**
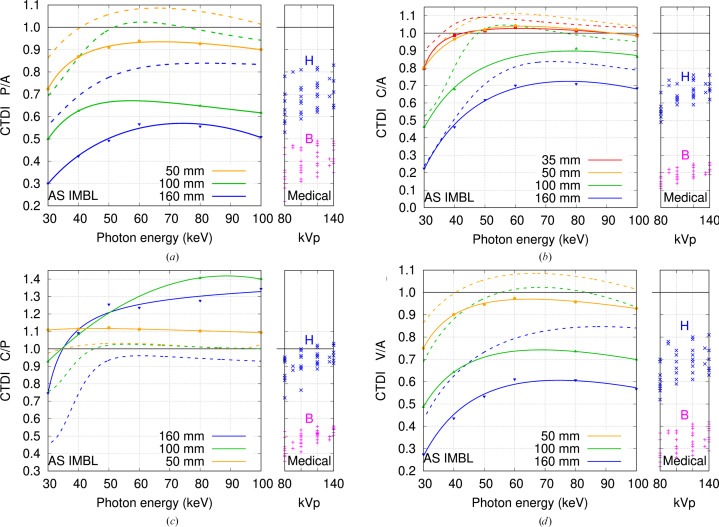
CTDI ratios for 4.0 T without (dotted) and 3.0 T with beam shaping filter (solid lines and points), versus medical CT with head (H) and body (B) bow-tie filters: (*a*) peripheral, (*b*) central to CTDI in air, (*c*) central to peripheral CTDI, and (*d*) volumetric to CTDI in air. For meaningful comparison, CTDI in air values are adjusted to include attenuation by a minimum of 10 mm PMMA filter thickness.

CTDI results are presented in Fig. 7 ▸ for a 3.0 T wiggler field normalized to the incident dose rate measured in air, and in Fig. 7 ▸(*c*) showing the ratio of central to peripheral CTDI. Results compare ratios of dose rate for 3.0 T with beam shaping filter (this study), to previously measured ratios without filters for 4.0 T and involving a similar wiggler spectrum. For a more meaningful comparison between these measurements, the 3.0 T CTDI measurements in air are adjusted to include attenuation by the minimum 10 mm path length through PMMA, amounting to 0.70–0.82 at 30–100 keV. Smooth curves are ratios of third to second degree polynomials fitted using *gnuplot*. Two data points are absent as the 100 mm diameter phantom was not fully secured to the rotation stage. Rapid counter rotation between scans created strong vibration that shifted this phantom out of the collimated beam. The peripheral measurements at 40 and 60 keV were rejected due to truncation. The lighter phantoms were secured with a reusable putty like adhesive, whilst the 160 mm diameter phantom was held in place by its own weight.

## 4. Discussion

For the ideal image receptor (with no additive sources of noise), the relationship between incident dose in air and CT image quality expressed as the noise-to-signal ratio NSR and spatial resolution *R* (Barrett *et al.*, 1976 ▸; Webb, 1988 ▸) can be written as 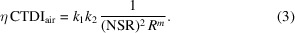
In this expression, *R* is set to the reconstructed voxel size and slice thickness, η is the detection efficiency, and exponent *m* equals 4 for filtered back projection (FBP) or 3 for iterative reconstruction methods. The difference in exponent *m* is a consequence of FBP removing the 1/*r* blurring of pure back projection (where *r* is the distance from the rotation axis) by using a frequency space ramp that also amplifies reconstructed image noise. Constant *k*_1_ depends on definitions of spatial resolution, noise and dose, whilst *k*_2_ is determined by the beam quality, scanned object size, density and composition. Thus for FBP, halving NSR for the same spatial resolution increases the dose fourfold, whilst halving spatial resolution for the same NSR requires a 16-fold increase in radiation dose. Synchrotron source brightness enables CT studies to be conducted at fine spatial resolution accompanied by increases in dose to the sample as per equation (3). For biological samples, the CTDI ratios presented in Fig. 7 ▸ can be used to scale the product of dose rate in air and exposure time, to estimate central, peripheral and volumetric CTDI.

The energy window offered by the IMBL is bounded at the low end by the selection of in-beam filters, whilst the upper limit is controlled by the choice of wiggler field and reduced efficiency of the DCLM (Fig. 3 ▸). Mechanical movement of the DCLM imposes a hard limit near 17 keV, whilst previous transmission measurements for 4.0 T operation found that the 20 keV beam contained only harmonic radiation. Added filter set F1 in conjunction with the DCLM and about 25 m of air offers the minimum amount of attenuation to remove photon energies below 20 to 24 keV where transmission is 0.1 to 1.0%, respectively. Filter set F2 is the preferred option as additional aluminium prevents heat from reaching the beamline components and, in conjunction with the DCLM, removes photon energies below 23 to 28 keV. Filter set F3 introduces additional copper to remove photon energies below 56 to 69 keV for 0.1 to 1.0% transmission, respectively. CTDI measurements in air (Fig. 6 ▸) show an upper limit that is close to 80 keV for a wiggler field of 1.4 T, approximately 100 keV for 2.0 T, and extending beyond 100 keV for 3.0–4.2 T.

The dynamic range for the Ruby camera restricted measurements to ray-sums of less than 7, which is insufficient to partially remove the third harmonic and reveal higher order contributions. Harmonic radiation can introduce systematic errors to quantitative CT, manifested as spatial variations in the reconstructed pixel values representing attenuation coefficients. This is tempered by the image receptor detection efficiency which decreases at higher photon energies, and by energy weighting that emphasizes higher energy photons. Results for the Ruby camera and mammography screen presented in Fig. 4 ▸ and Table 2 ▸ show reduced harmonic contribution for lower wiggler field strengths. Fig. 5 ▸ predicts measured ray-sum errors for biological samples, approximated by water and aluminium, representing attenuation by soft tissue and cortical bone, respectively. Harmonic radiation is responsible for ray-sum systematic errors, and these increase with atomic number for lower energies and thicker samples. Harmonic content is reduced for lower wiggler field allowing quantitative CT with thicker specimens as shown in Fig. 5 ▸. If a systematic error of −0.5% is deemed to be acceptable, then at 30 keV the maximum ray-sum for soft tissue is increased from 3 at 3.0 T to 5 at 1.4 T, whilst for cortical bone the increase is 1.5 at 3.0 T to 3 at 1.4 T. For the Ruby camera, the ratio of dark signal to incident signal is 0.2%, reducing the upper thickness limit in Fig. 1 ▸(*a*) to ray-sums of 4.

Dosimetry measurements in air for operation at the DCLM rocking curve peak are summarized in Table 3 ▸. A reduction in wiggler field strength reduces the dose rate and shifts the available spectrum towards lower energies. The lowest energy for quantitative CT studies can be inferred from the high energy limits shown Fig. 6 ▸, whereby energies above 26 keV for 1.4 T and above 33 keV for 2.0 T are without significant harmonics. These findings are specific to the IMBL source comprising SCMPW and DCLM.

Fig. 7 ▸ presents CTDI ratios for 10 mm axial beam collimation, comparing measurements at 3.0 T with beam shaping filters against previous measurements at 4.0 T without these devices. These results may be applied to CT studies at other beamlines. Medical CT with 80–140 kVp tungsten radiation, filtered by at least 10 mm aluminium or equivalent, produces beam qualities similar to 65–80 keV mono-energetic radiation. CTDI ratios for 80–140 kVp medical head CT are similar to those for mono-energetic CT at energies 50–100 keV. The CTDI ratios relative to air are reduced for larger diameter objects and at lower photon energies due to increased beam attenuation. Our phantoms fall into two classes: ‘small’ with diameters 50 mm or less versus ‘large’. The peripheral to air ratio in Fig. 7 ▸(*a*) is reduced with beam shaping filters by 5–10% for small and by 20–30% for large phantoms. The central to air ratio in Fig. 7 ▸(*b*) is near unity for small samples, where beam shaping filters deliver a 5% reduction. For larger samples, these ratios are near-constant above 60 keV, and reduced by 10% with shaping filters. Ratios are far lower below 60 keV, and shaping filters deliver no change at 30 keV. The ratio for central to peripheral CTDI in Fig. 7 ▸(*c*) is ideally near unity, representing a uniform dose profile across the scanned object. Without beam shaping the ratio is near unity, except for large samples below 50 keV, where CTDI at the centre is reduced due to attenuation. Beam shaping filters reduce the peripheral CTDI, leading to the observed increases for the central to peripheral CTDI ratio. The key result is Fig. 7 ▸(*d*), showing the ratios of volumetric CTDI to values in air. The ratio is near unity for small, weakly attenuating phantoms, and less for larger phantoms. Beam shaping filters reduce the ratio by about 10% for small objects and by 20–30% for the larger samples considered here with diameters up to 160 mm.

## 5. Conclusions

Synchrotron CT can deliver a substantial radiation dose to the scanned object and the image receptor. Contributing factors include source brightness, the quest for higher spatial resolution accompanied by less than ideal detection efficiency, extended CT scan times, and the relationship between dose and image quality summarized by equation (3). This study examined two CT dose reduction strategies: adjusting the wiggler spectrum via the field strength, and spatial modulation of the lateral beam profile for CT studies with biological samples of diameter 35–160 mm.

The key considerations are the range of available mono-energetic beam energies, the influence of harmonic radiation, and the available flux quantified here as the dose rate in air. Attenuation by the beamline components is responsible for the low energy cut-off at 23–28 keV for 0.1–1% transmission due to the combined effects of filter set F2, a path length of 25 m through air and at least 2.0 mm of silicon for the DCLM. Beam quantity and quality were measured for wiggler magnetic fields below the ‘standard’ 4.0 T, finding that the influence of harmonic radiation is reduced for measurements at 25 and 30 keV. This study quantifies how wiggler field strength controls the available spectrum, dose rate and harmonic content passed by the DCLM. The maximum available energy extends beyond 100 keV for 3.0–4.2 T, to 100 keV for 2.0 T and to 80 keV for 1.4 T. Relative to 3.0 T wiggler operation, the mean dose rate in air is reduced to 12% at 2.0 T and to 4.0% at 1.4 T, enabling CT imaging at reduced dose rate, also delaying radiation damage to image receptors. Lower wiggler fields reduce the influence of harmonic radiation, whereby, for filter set F2, beam energies above 26 keV at 1.4 T and beam energies above 33 keV at 2.0 T are without significant harmonic radiation content.

The outcomes of this work can be extended to other beamlines. The energy window for mono-energetic CT with soft tissue like samples of different sizes is shown in Fig. 1 ▸. Filter transmission (Fig. 1 ▸), harmonic content (Figs. 4 ▸ and 5 ▸) and source brightness as a function of operational parameters (Fig. 6 ▸) are specific to each beamline. Dosimetry estimates for mono-energetic CT without and with beam shaping filters can use the ratios presented in Fig. 7 ▸ combined with site specific estimate for the incident CTDI in air, either measured directly or estimated via the product of air kerma rate at the isocentre and exposure duration.

Beam shaping filters placed upstream of the sample stage aim to deliver near-uniform transmitted intensity, with the added benefit of reducing the absorbed dose to shallow regions of the sample. In the absence of the sample, the ‘empty’ image profile is no longer ‘flat’ but ‘convex’, requiring a shorter frame integration time to avoid saturation and the acquisition of more frames to compensate for this change. The axial collimation, scan length, helical pitch and CTDIvol are used by medical CT dose calculators (Stamm & Nagel, 2002 ▸; IMPACT, 2011 ▸) to estimate individual organ doses and the whole body effective dose. Without access to CT dose calculators for small animals, the CTDI ratios peripheral-to-air can be used to estimate absorbed dose to organs near the surface and central-to-air for the deeper organs. Beam shaping filters offer radiation protection to the sample by reducing the peripheral and volumetric CTDI by about 10% for small objects and 20–30% for the larger samples considered here. The image receptor is also protected by attenuating the primary beam during the CT scan to near homogeneous dose rate across the entire field of view.

## Figures and Tables

**Table 1:** Location *z*, composition and path length *t* for in-beam filters and other materials (excluding vacuum) between the wiggler source and experimental hutches; 1B and 2B for radiobiology studies and 3B for imaging and CT studies

Hutch	*z* (m)	Component			Material	*t* (mm)
1A	10	Window			C (diamond)	0.6
1A	14.7	*In vacuo* filter sets				
		F1	F2	F3	C (graphene)	0.45
		F1	F2	F3	C (graphite)	21.2
		–	F2	F3	Al	2.83
		–	–	F3	Cu	2.83
1A	16.2	DCLM			Si	2 × 1.0
1B to 2B	20.4 to 40.3	Path through hutches			Air	19900
2A	30.7	Window			Be	0.35
2B	31.5	Window			Be	0.35
3A	135.8	Window			Be	2.0
3B	135.8 to 140.3	Air path to 3B centre			Air	4500

**Table 2:** Fit results for third harmonic contribution *f* to total intensity (1 + *f*)

*E* (keV)	*B* (T)	*f* (%)
25	1.4	0.83
25	2.0	3.61
30	1.4	0.22
30	2.0	0.30
30	3.0	0.66
60	3.0	0.35

**Table 3:** Measured dose rates in air (mGy s^−1^), the mean over 25–100 keV and peak value at photon energy *E*_P_

*B* (T)	25 keV	100 keV	Mean	Peak	*E*_P_ (keV)
3.0	21.7	15.2	62.1	145	45
2.0	2.04	0.51	7.7	19.2	42
1.4	1.03	–	2.7	6.35	38
